# CCL5 promotes VEGF-C production and induces lymphangiogenesis by suppressing miR-507 in human chondrosarcoma cells

**DOI:** 10.18632/oncotarget.9213

**Published:** 2016-05-06

**Authors:** Li-Hong Wang, Chih-Yang Lin, Shih-Chia Liu, Guan-Ting Liu, Yen-Ling Chen, Jih-Jung Chen, Chia-Han Chan, Ting-Yi Lin, Chi-Kuan Chen, Guo-Hong Xu, Shiou-Sheng Chen, Chih-Hsin Tang, Shih-Wei Wang

**Affiliations:** ^1^ Department of Orthopedics, Dongyang People's Hospital, Wenzhou Medical University, Dongyang, China; ^2^ Graduate Institute of Basic Medical Science, China Medical University, Taichung, Taiwan; ^3^ Department of Orthopaedics, Mackay Memorial Hospital, Taipei, Taiwan; ^4^ Department of Fragrance and Cosmetic Science, College of Pharmacy, Kaohsiung Medical University, Kaohsiung, Taiwan; ^5^ Department of Pharmacy, Tajen University, Pingtung, Taiwan; ^6^ Department of Medicine, Mackay Medical College, New Taipei City, Taiwan; ^7^ Department of Pathology, Mackay Memorial Hospital, Taipei, Taiwan; ^8^ Department of Urology, National Yang-Ming University School of Medicine, Taipei, Taiwan; ^9^ Division of Urology, Taipei City Hospital Renai Branch, Taipei, Taiwan; ^10^ Department of Pharmacology, School of Medicine, China Medical University, Taichung, Taiwan; ^11^ Department of Biotechnology, College of Health Science, Asia University, Taichung, Taiwan

**Keywords:** CCL5, VEGF-C, lymphangiogenesis, miR-507

## Abstract

Chondrosarcoma is the second most frequently occurring type of bone malignancy that is characterized by the distant metastasis propensity. Vascular endothelial growth factor-C (VEGF-C) is the major lymphangiogenic factor, and makes crucial contributions to tumor lymphangiogenesis and lymphatic metastasis. Chemokine CCL5 has been reported to facilitate angiogenesis and metastasis in chondrosarcoma. However, the effect of chemokine CCL5 on VEGF-C regulation and lymphangiogenesis in chondrosarcoma has largely remained a mystery. In this study, we showed a clinical correlation between CCL5 and VEGF-C as well as tumor stage in human chondrosarcoma tissues. We further demonstrated that CCL5 promoted VEGF-C expression and secretion in human chondrosarcoma cells. The conditioned medium (CM) from CCL5-overexpressed cells significantly induced tube formation of human lymphatic endothelial cells (LECs). Mechanistic investigations showed that CCL5 activated VEGF-C-dependent lymphangiogenesis by down-regulating miR-507. Moreover, inhibiting CCL5 dramatically reduced VEGF-C and lymphangiogenesis in the chondrosarcoma xenograft animal model. Collectively, we document for the first time that CCL5 induces tumor lymphangiogenesis by the induction of VEGF-C in human cancer cells. Our present study reveals miR-507/VEGF-C signaling as a novel mechanism in CCL5-mediated tumor lymphangiogenesis. Targeting both CCL5 and VEGF-C pathways might serve as the potential therapeutic strategy to block cancer progression and metastasis in chondrosarcoma.

## INTRODUCTION

Human chondrosarcoma is the second most common sarcoma arising in bone malignancy which chiefly occurs in adults over 40 years of age [1]. Chondrosarcoma has been characterized as the aggressive and pathologically diverse malignant tumor with poor disease progression [2]. To date, surgical resection remains the primary treatment for chondrosarcoma, since conventional radiotherapy and chemotherapy are mostly ineffective. The relapse ususally occurs after surgical resection because of the potential for metastatic propensity. The need for a specific targeted therapy to impede the metastasis of chondrosarcoma remains urgent [3].

Metastasis is the leading cause of cancer mortality worldwide. The metastatic spread of tumor cells to sentinel lymph nodes represents the first step of tumor dissemination in most human cancers [4]. Lymphangiogenesis, the formation of new lymphatic vessels from preexisting ones, is crucial for the development of tumor metastasis [5]. Tumors can actively promote the formation of lymphatic vessels through secretion of lymphangiogenic factors, and that tumor lymphangiogenesis has been implicated in the correlation with lymph node metastasis in many types of human cancer [6]. Vascular endothelial growth factor-C (VEGF-C) is the best-characterized lymphangiogenic factor, acting predominantly via VEGF receptor-3 (VEGFR-3) that is specifically expressed by lymphatic endothelial cells (LECs). The activation of VEGF-C/VEGFR-3 axis is responsible for LECs survial, proliferation, migration and tube formation during lymphangiogenic process [7]. Recent studies have revealed that VEGF-C production by tumor cells is recognized as the chief promoter of tumor-associated lymphangiogenesis and lymphatic metastasis [5]. Moreover, clinical evidences suggest the existence of a relationship between tumor expressing VEGF-C and the disease progression of cancer in various tumor types, including melanoma, pancreatic, breast, colorectal and lung cancer [8–12]. Blockade of tumor-induced lymphangiogenesis has been shown to markedly suppress cancer metastasis. Therefore, the identification of mechanisms underlying VEGF-C-mediated lymphangiogenesis is essential for developing novel prognostic and therapeutic strategies in the treatment of cancer [13, 14].

MicroRNAs (miRNAs) are small non-protein-coding RNAs that function as endogenous negative gene regulators by interfering with the translation or stability of target transcripts [15]. They control gene expression by binding to the 3′untranslated region (3′UTR) of mRNA through complementary base pairing. Dysfunctions of miRNAs are frequently found in malignancies, including chondrosarcoma [16]. Increasing studies have focused on the role of miRNAs in cancer progression and metastasis. miRNAs have been proposed to intervene numerous functions of cancer cell, including survival, apoptosis, autophagy, migration, invasion, angiogenesis and lymphangiogenesis [17]. Several investigations demonstrate that miRNAs inhibit lymphangiogenesis and tumor dissemination through the dysregulation of miR/VEGF-C signaling [18, 19]. miR-128 has been reported to inhibit lymphangiogenesis in human non-small cell lung cancer by directly suppressing VEGF-C expression [20]. miR-206 also abrogates the expression and secretion of VEGF-C, and subsequently inhibits tumor lymphangiogenesis in pancreatic cancer [21]. Furthermore, miR-101 has been documented to suppress migration and invasion via negatively regulating VEGF-C expression in bladder cancer and cholangiocarcinoma cells, respectively [22]. However, the role of miRNA in regulating VEGF-C expression in human chondrosarcoma cells is poorly understood.

CCL5 is widely established as an inflammatory chemokine secreted by many cell types including activated T cell, macrophage, endothelial cell, stromal cells and cancer cells [23, 24]. CCL5 has been demonstrated to play the important role in many chronic inflammatory diseases. Compelling evidences indicate that CCL5 is associated with tumourigenesis and metastasis in several types of cancer [25]. We previously reported that CCL5 enchances cell migration through activation of matrix metalloproteinase-3 (MMP-3) and promotes VEGF-A-dependent tumor angiogenesis in human chondrosarcoma [26–28], implying that CCL5 is involved in the metastasis of chondrosarcoma. However, it is still not well-recognized whether CCL5 increases VEGF-C expression to facilitate tumor-associated lymphangiogenesis in human chondrosarcoma. In this study, we investigated the relationship of CCL5 with VEGF-C-mediated lymphangiogenesis, and evaluated the involvement of miRNA in human chondrosarcoma cells.

## RESULTS

### Clinical significance of CCL5 and VEGF-C expression in specimens from patients with chondrosarcoma

**Figure 1:**
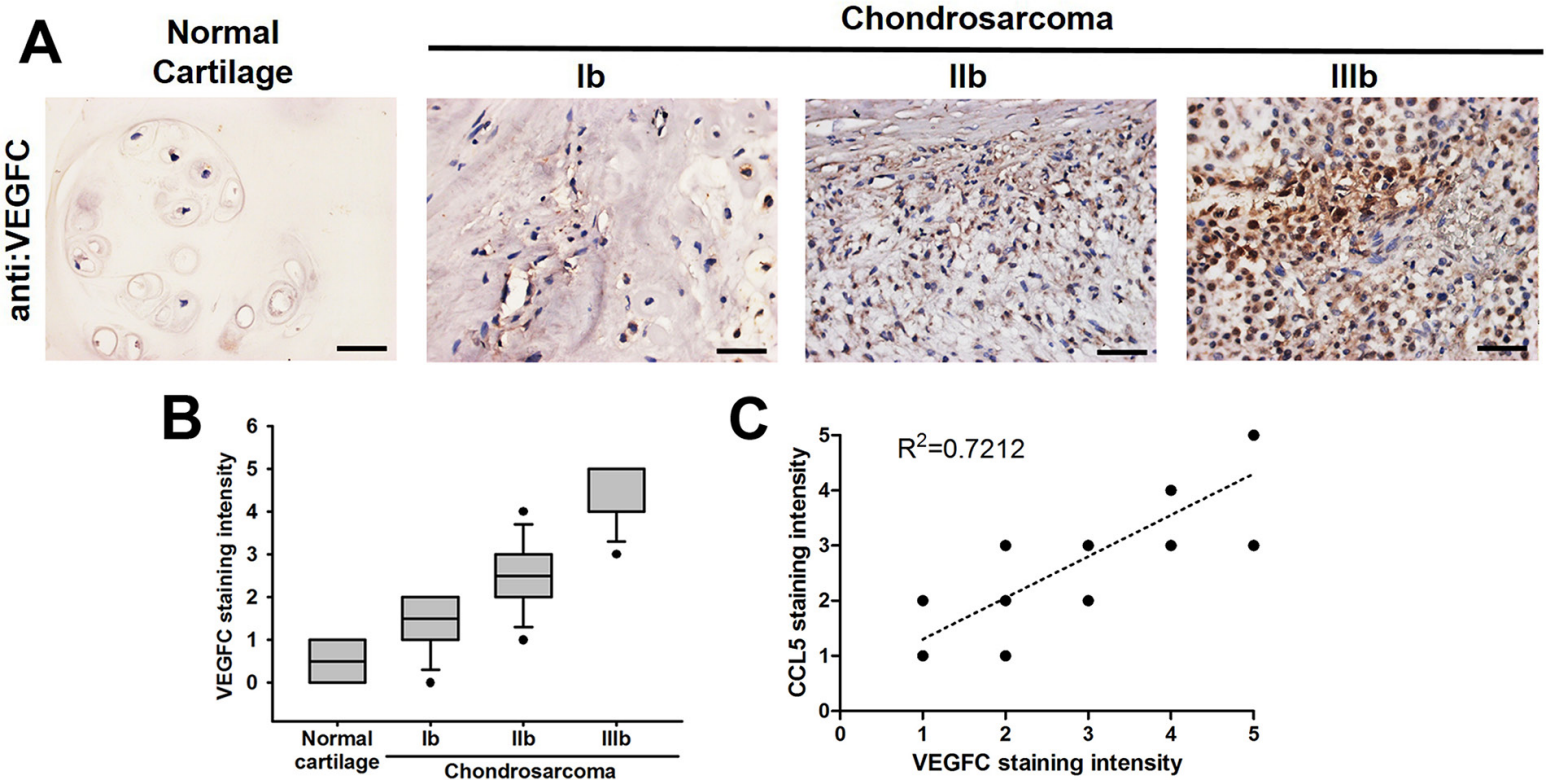
Clinical significance of CCL5 and VEGF-C expression in specimens of chondrosarcoma patients **A.** Immunohistochemistry of VEGF-C expression in human normal cartilage and chondrosarcoma tissues. **B&C.** The quantitative data and correlation between CCL5 and VEGF-C as well as tumor stage. The representative specimens were shown, and the staining intensity was ranked into five groups (1-5) according to the histologic scoring. The correlation analyses were performed by Spearman rank correlation test (R^2^ > 0.5).

Our previous studies have shown that chemokine CCL5 facilitates tumor angiogenesis in human chondrosarcoma [27, 28]. We indicated that the expression of CCL5 is highly correlated with tumor stage according the IHC analysis of human chondrosarcoma tissues. To characterize the role of CCL5 in tumor lymphangiogenesis of chondrosarcoma, we first analyzed the expression profile of VEGF-C in specimens of chondrosarcoma patients. As shown in Figure 1A, the expression of VEGF-C was higher in tumor specimens than in normal tissues. Accordingly, the high level of VEGF-C expression correlated significantly with tumor stage (Figure 1B). The quantitative data also showed the high positive relationship between the expression of CCL5 and VEGF-C in human chondrosarcoma patients (Figure 1C). In addition, we examined the mRNA expression of CCL5, CCL5 receptors and VEGF-C between normal caritlage and chondrosarcoma. The expression of CCL5 and VEGF-C in chondrosarcoma patients was obvioustly higher than that in normal cartilage (Supplementary Figure 1A&1B). The mRNA levels of CCR5 and GPR75 were higher in tumor specimens than in normal tissues (Supplementary Figure 1C&1D). These results suggest that chemokine CCL5 and its receptors are strongly associated with VEGF-C expression and tumor progression of human chondrosarcoma.

### CCL5 promotes VEGF-C-dependent lymphangiogenesis in human chondrosarcoma cells

**Figure 2:**
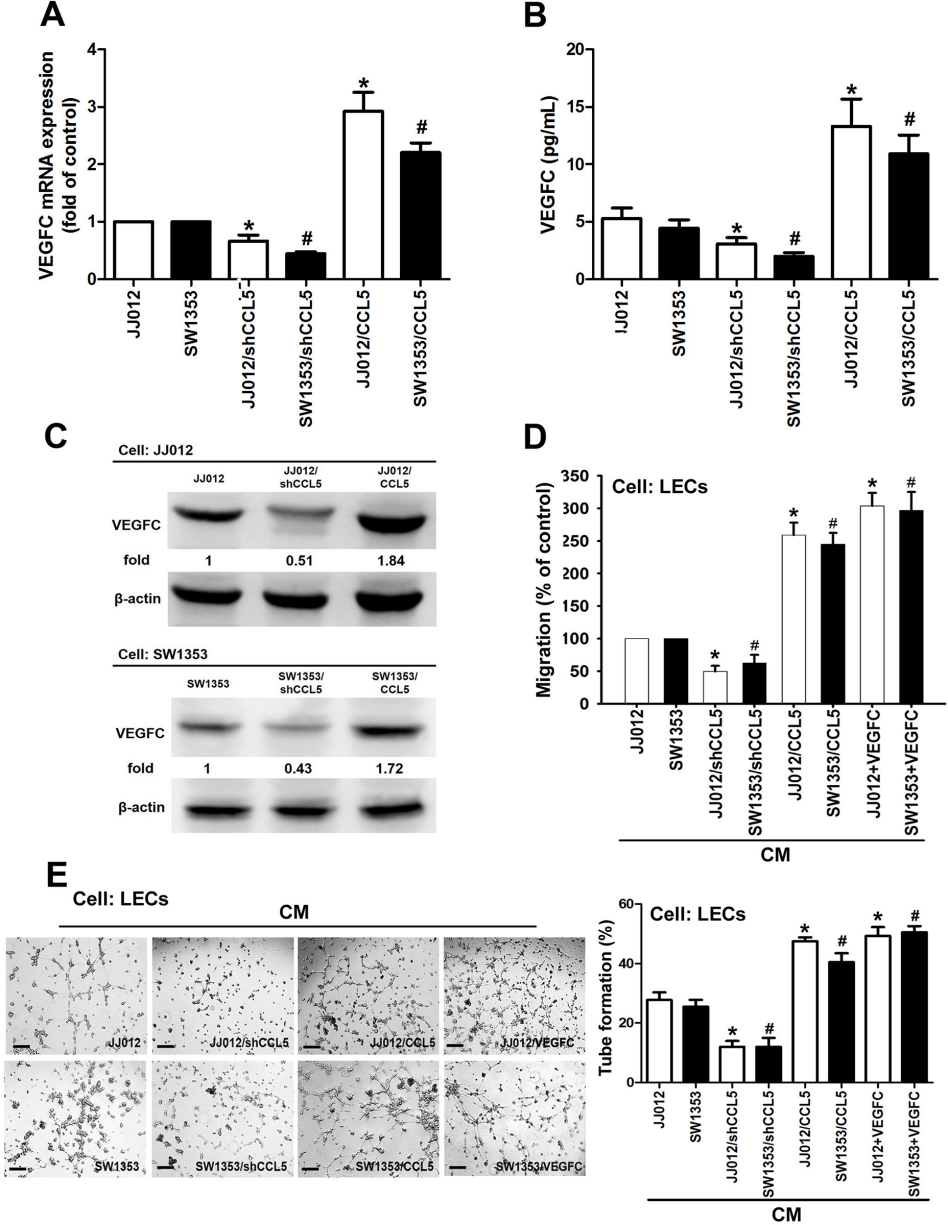
CCL5 promotes lymphangiogenesis by VEGF-C production in human chondrosarcoma cells **A-C.** The JJ012/vector, JJ012/shCCL5, JJ012/CCL5, SW1353/vector, SW1353/shCCL5 and SW1353/CCL5 cells were cultured for 24 h, and VEGF-C expression was examined by qPCR, ELISA, and Western blot (*n* = 4-6). **D&E.** The medium was collected as CM, then applied to LECs for 6 h treatment. Then, cell migration and capillary-like structure formation in human LECs were examined by Transwell migration and tube formation assay. The CM of JJ012/vector and SW1353/vector cells were treated with VEGF-C (25 ng/ml), then used to induce LECs migration and tube formation (*n* = 4-6). Results are expressed as the mean ± SE. *, *p* < 0.05 compared with JJ012 control; #, *p* < 0.05 compared with SW1353 control.

To clarify direct relationship between CCL5 and VEGF-C in lymphangiogenic process, CCL5 stable transfectant in human chondrosarcoma cells were established (Supplementary Figure 2). After proper selection by antibiotics, CCL5-overexpressed clone (JJ012/CCL5 and SW1353/CCL5), CCL5-knockdowned clone (JJ012/shCCL5 and SW1353/shCCL5) and vector control (JJ012/vector and SW1353/vector) cells were generated to investigate the effect of CCL5 on VEGF-C expression and lymphangiogenesis. The results showed that CCL5 overexpression manifestly increased VEGF-C mRNA and protein levels, and the secretion of VEGF-C (Figure 2A-2C). To elucidate the interaction of CCL5 with it specific receptor CCR5, the selective CCR5 antagonists (maraviroc and DAPTA) were used in CCL5-overexpressed cells. Maraviroc and DAPTA markedly attenuated CCL5-induced VEGF-C mRNA expression, implying that CCL5 promotes VEGF-C expression through chemokine receptor CCR5 in an autocrine manner (Supplementary Figure 3). We then examined whether CCL5-dependent VEGF-C expression induced lymphangiogenesis using an *in vitro* LECs model. Incubation of human LECs with conditioned medium (CM) from CCL5-overexpressed cells significantly promoted migration and tube formation of LECs (Figure 2D&2E). The human recombinant VEGF-C was used as the positive control to induce tube formation of LECs. In contract, shRNA-mediated knockdown of CCL5 notably inhibited VEGF-C expression and secretion in human chondrosarcoma cells, and subsequently suppressed migration and tube formation in human LECs (Figure 2). These results indicate that CCL5/CCR5 axis enhances VEGF-C production and lymphangiogenesis in human chondrosarcoma.

### CCL5 enhances VEGF-C production and lymphangiogenesis by down-regulating miR-507

**Figure 3:**
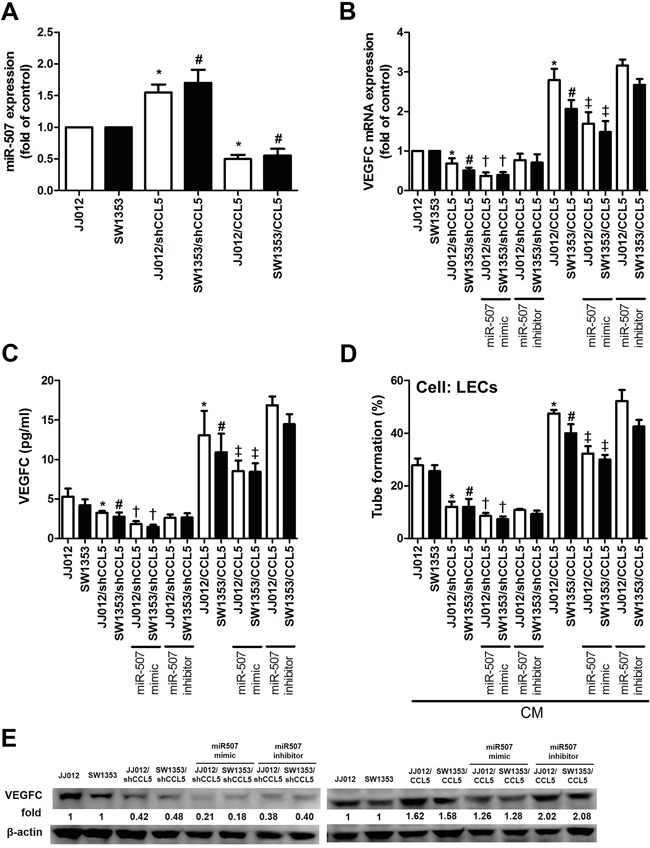
CCL5 promotes VEGF-C-dependent lymphangiogenesis by suppressing miR-507 The JJ012/vector, JJ012/shCCL5, JJ012/CCL5, SW1353/vector, SW1353/shCCL5 and SW1353/CCL5 cells cultured for 24 h, and miR-507 expression was examined by qPCR **A.** In addition, cells were transfected with miR-507 mimic or inhibitor for 24 h, and VEGF-C expression was examined by qPCR **B.** ELISA **C.** and Western blot **E.** (*n* = 5-7). The medium was collected as CM and then applied to LECs for tube formation assay **D.** (*n* = 5). Results are expressed as the mean ± SE. *, *p* < 0.05 compared with JJ012 control; #, *p* < 0.05 compared with SW1353 control; †, *p* < 0.05 compared with CCL5-knockdowned group; ‡, *p* < 0.05 compared with CCL5-overexpressed group.

Emerging studies have indicated that miRNAs are important regulators of VEGF-C expression and tumor lymphangiogenesis in cancer progression [17, 18]. We next performed miRNome microRNA Profilers QuantiMir™ kit to elucidate miRNA differential expression in CCL5-overexpressed chondrosarcoma cells. This kit contained 384 human miRNA primer, we analyzed 33 metastatic miRNA and found the miR-507 was the most downregulated in response to CCL5 overexpression (Supplementary Figure 4). We further integrated the miRNA screening results with open source software (www.TargetScan.org and www.microrna.org), and found that the 3′-UTR of VEGF-C mRNA harbors potential binding sites for miR-507. We confirmed miR-507 expression using quantitative real-time PCR (qPCR) in both CCL5-overexpressed and CCL5-knockdowned cells. As shown in Figure 3A, the miR-507 was downregulated in JJ012/CCL5 and SW1353/CCL5 cells, and upregulated in JJ012/shCCL5 and SW1353/shCCL5 cells. To explore the role of miR-507 in CCL5-induced VEGF-C production and lymphangiogenesis, we transiently transfected the miR-507 mimic or miR-507 inhibitor into CCL5-overexpressed cells and CCL5-knockdowned cells, respectively. The data showed that VEGF-C production and LECs tube formation were obviously reduced by the miR-507 mimic in JJ012/shCCL5 and SW1353/shCCL5 cells. In addition, the miR-507 mimic clearly diminished the CCL5-induced VEGF-C production and LECs tube formation in JJ012/CCL5 and SW1353/CCL5 cells (Figure 3B-3E).

**Figure 4:**
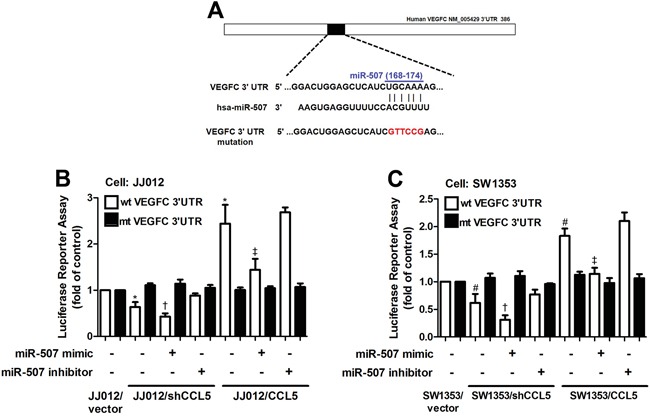
The miR-507 inhibits VEGF-C expression through binding to the 3′UTR of human *VEGF-C* **A.** Schematic representation of the 3′UTR of the human VEGF-C containing a miR-507 binding site. **B&C.** Cells were cotransfected with miR-507 mimic or inhibitor and wt-VEGFC-3′UTR or mt-VEGFC-3′UTR plasmid for 24 h, and the relative luciferase/renilla activities were measured by luciferase reporter assay (*n* = 5). Results are expressed as the mean ± SE. *, *p* < 0.05 compared with JJ012 control; #, *p* < 0.05 compared with SW1353 control; †, *p* < 0.05 compared with CCL5-knockdowned group; ‡, *p* < 0.05 compared with CCL5-overexpressed group.

In order to verify whether miR-507 regulates the 3′UTR of *VEGF-C*, we constructed luciferase reporter vectors harboring wildtype 3′UTR of *VEGF-C* mRNA (wt-VEGFC-3′UTR) and vector containing mismatches in the predicted miR-507 binding site (mt-VEGFC-3′UTR) and transfected these vectors into CCL5-overexpressed cells, CCL5-knockdowned cells and control cells. We found that cotransfection with the miR-507 mimic inhibited luciferase activity in the wt-VEGFC-3′UTR plasmid but not in the mt-VEGFA-3′UTR plasmid (Figure 4). Taken together, these findings demonstrate that miR-507 directly represses VEGF-C expression via binding to 3′UTR of the human *VEGF-C* gene.

### CCL5 boosts tumor-associated lymphangiogenesis by down-regulating miR-507

**Figure 5:**
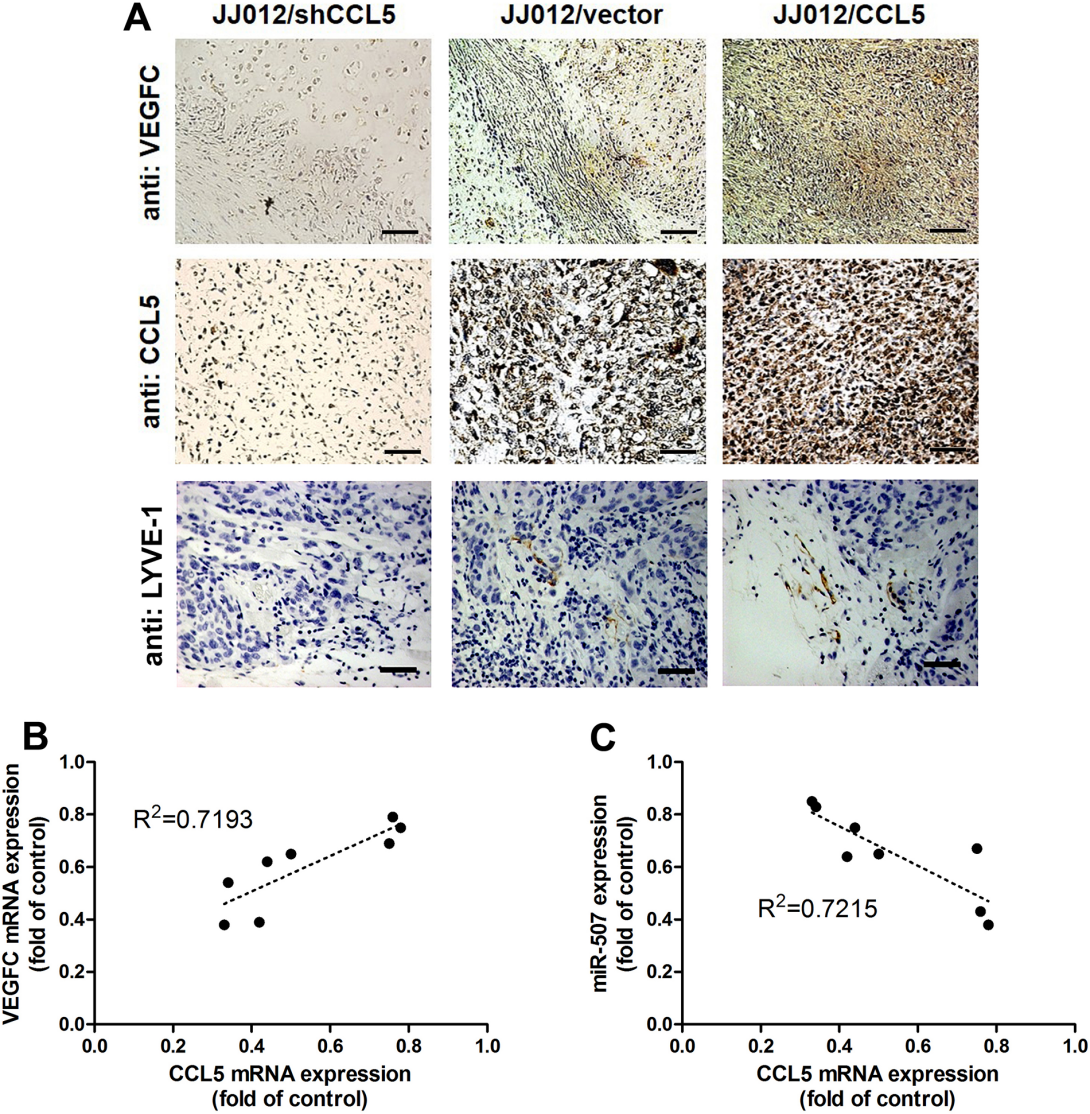
CCL5 promotes tumor lymphangiogenesis by suppressing miR-507 *in vivo* The JJ012/vector, JJ012/shCCL5 and JJ012/CCL5 cells were mixed with Matrigel and injected into flank sites of mice (*n* = 8-10) for six weeks. Then, the xenografted tumors were excised and stained with CCL5, VEGF-C and LYVE-1 by IHC **A.** The correlation between CCL5/VEGF-C **B.** and CCL5/miR-507 **C.** in tumor tissues was analyzed by qPCR and Spearman rank correlation test (*n* = 8) (R^2^ > 0.5).

Herein, we investigated whether CCL5 promoted tumor lymphangiogenesis *in vivo*. To validate the effect and targeting miRNA of CCL5 on tumor lymphangiogenesis, we analyzed the expression of CCL5, VEGF-C and miR-507 in tumor tissues which were excised from chondrosarcoma xenograft mice. Immunohistochemical analysis revealed that overexpression of CCL5 increased the expression of CCL5 and VEGF-C, whereas knockdown of CCL5 decreased the expression of CCL5 and VEGF-C in mouse chondrosarcoma tissues (Figure 5A). The quantitative data exhibited the positive correlation between CCL5 and VEGF-C, and the negative correlation between CCL5 and miR-507 using qPCR analysis in tumor specimens form animals (Figure 5B&5C). Furthermore, LYVE-1 is the specific marker of lymphatic vessels in tumor lymphangiogenesis. As shown in Figure 5A, the number of lymphatic vessels, stained with anti-LYVE-1, was reduced by knockdown of CCL5, and promoted by overexpression of CCL5. Therefore, we suggest that CCL5 promotes VEGF-C-dependent tumor-associated lymphangiogenesis by down-regulating miR-507 *in vivo*.

## DISCUSSION

**Figure 6:**
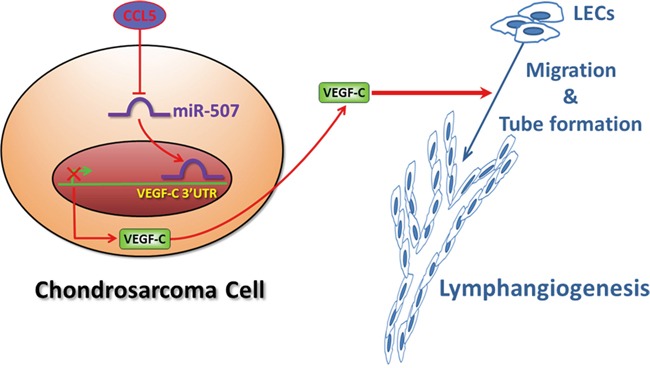
Schematic diagram summarizes the mechanism of CCL5-mediated tumor lymphangiogenesis in human chondrosarcoma CCL5 promotes VEGF-C production by downregulating miR-507 in human chondrosarcoma cells, and subsequently induces lymphangiogenesis of human LECs.

Although chondrosarcoma is a relatively rare human cancer, the notorious aggressiveness of chondrosarcoma is due to its metastatic potential and poor prognosis [3]. Lymphngiogenesis is an indispensable step for cancer metastasis, facilitating cancer development by the generation of new lymphatic vessels that serve as conduits for tumor dissemination to lymph nodes and beyond [4]. Accumulating evidences demonstrate that increased level of VEGF-C promotes tumor relapse and poor prognosis, and thus VEGF-C represents a potential target for preventing lymphatic metastasis [5, 13]. In the current study, we indicate the clinical significance of CCL5 and VEGF-C expression in specimens of chondrosarcoma patients. We reveal that CCL5 increases the production of lymphangiogenic factor VEGF-C by down-regulating miR-507 in chondrosarcoma cells, and thereby promotes tumor lymphangiogenesis in chondrosarcoma microenvironment (Figure 6). Recent studies have shown that chemokine/chemokine receptor system is directly or indirectly involved in promoting tumor lymphangiogenesis [5]. CXC chemokine ligand 12 (SDF-1) has been indicated to directly promote lymphangiogenesis in human LECs through the chemokine receptor CXCR4 [29]. Addtionally, CCL21/CCR7 chemokine axis regulates the expression and secretion of VEGF-C in human breast cancer cells, and contributes to LECs lymphangiogenesis and breast cancer-induced lymphangiogenesis [30]. Herein, we point out that CCL5/CCR5 axis induces VEGF-C-dependent lymphangiogenesis in human chondrosarcoma. Our study is the first to report that chemokine CCL5 induces lymphangiogenesis by the induction of VEGF-C in human cancer cells.

Chemokines can be produced by tumor cells and/or by nonmalignant stromal cells in the microenvironment, and dictate tumor development by stimulating angiogenesis and lymphangiogenesis, which are the important routes for cancer metastasis [31]. Here, we investigated the effect of chemokine CCL5 on VEGF-C production and tumor lymphangiogenesis both *in vitro* and *in vivo.* We found that CCL5 increased the expression and secretion of VEGF-C in human chondrosarcoma cells, and subsequently induced migration and tube formation of human LECs. Otherwise, CCL5 knockdown dramatically suppressed VEGF-C-induced lymphangiogenesis. Moreover, we showed that CCL5 and VEGF-C display a positive correlation in the chondrosarcoma xenograft model. CCL5 knockdown diminished the expression of lymphangiogenesis marker LYVE-1 and VEGF-C *in vivo.* CCL5 serves as a potent positive-regulator of tumor lymphangiogenesis in human chondrosarcoma. Previously, we have demonstrated that CCL5 promotes tumor angiogenesis by VEGF-A production in human chondrosarcoma [27, 28]. These findings support the rationale that impeding CCL5 signal may inhibit both tumor angiogenesis and lymphangiogenesis, leading to impair cancer metastasis and the formation of an immunosuppressive microenvironment. Targeting CCL5 and CCR5 with antagonists will have potential therapeutic usage to restrict the disease progression of many cancers, particularly chondrosarcoma. CCR5 antagonists have been successfully used in patients with HIV infection [32]. Maraviroc and vicriviroc are CCR5 antagonists that exert promising efficacy against chemokine function and HIV entry. There are several lines of evidence suggesting possible clinical applications of CCR5 antagonists in cancer treatment [33, 34]. Importantly, inhibition of CCL5 production by cancer cell or by the tumor microenvironment may represent an additional strategy to block cancer progression [25]. The development of novel therapeutic molecules (antibody, peptide, small molecule compound, etc.) aimed to repress the expression or secretion of CCL5 is needed to proceed in the future.

The lymphatic system is the primary pathway of metastasis for most human cancers [6]. The lymphatic endothelium, which comprises LECs, is a specialized endothelium and is distinct from the vascular endothelium [35]. Tumor lymphatic vessels serve as a pivotal route for metastatic cancer cells, due to their leaky nature and secretion of tumor-recruiting factors [7]. More understanding of molecular mechanisms underlying tumor lymphangiogenesis will provide new insights in the process of metastasis. However, the study of lymphangiogenesis has been impeded by the difficulties in the isolation and propagation of LECs from different organs [36, 37]. To overcome the above limitations, we used a “conditionally immortalized” line of human LECs, which transformed with the human telomerase reverse transcriptase (hTERT), and maintained their ‘lymphatic’ endothelial characteristics after repeated passages. This immortalized human LECs keep the ability to sprout, elongate, migrate and reorganize to form the capillary-like tube structure within 4–8 h, a process called tube formation, and this function of LECs represents the major process of lymphangiogenesis. In this study, we found that CM from CCL5-overexpressed cells profoundly stimulated tube formation of human LECs. On the contrary, knockdown of CCL5 suppressed CM-induced LECs tube formation. These results provide evidences that CCL5-mediated VEGF-C production induces lymphangiogenesis *in vitro*. Furthermore, we found that levels of CCL5 and VEGF-C in clinical specimens from patients with chondrosarcoma were correlated with tumor stage, implying that CCL5 may be a potential prognostic indicator for disease progression of human chondrosarcoma.

Small non-coding miRNAs, the average length of approximately 18 to 22 nucleotides, negatively regulate gene expression by either translational repression or mRNA cleavage through binding to complementary 3′UTR sequences of target mRNA [15, 17]. Deregulated biogenesis of miRNAs has been widely observed in human cancer [18]. Accumulating evidences further indicate that numerous miRNAs can impede cancer progression via direct suppression of VEGF-C. miR-27b, miR-101, miR-128, miR-206 and miR-1826 have been reported to inhibit tumor growth, lymphangiogenesis and metastasis by targeting VEGF-C in a variety of human cancer cells [20–22, 38–40]. Current study showed that CCL5 markedly repressed miR-507 expression in human chondrosarcoma cells *in vitro* and *in vivo*. Cotransfection of cells with miR-507 mimic abolished CCL5-induced VEGF-C production and LECs tube formation. Strikingly, we revealed that miR-507 directly inhibited VEGF-C protein expression through binding to the 3′UTR of the human *VEGF-C* gene, thereby negatively regulating VEGF-C-mediated lymphangiogenesis. miR-507 has been shown to suppress the NF-E2-related factor 2 (NRF2)-mediated oncogenic pathway by directly targeting NRF2 [41]. Moreover, administration of miR-507 alone or in combination with cisplatin significantly inhibits tumor growth in esophageal squamous cell carcinoma. To our knowledge, this study is first time to indicate that miR-507 negatively regulates VEGF-C expression. Importantly, we further demonstrate that CCL5 promotes VEGF-C-mediated tumor lymphangiogenesis by suppressing miR-507 in human chondrosarcoma. Thus, these findings provide information on the potential miRNA-based molecular diagnosis and treatment for VEGF-C-dependent tumor-associated lymphangiogenesis.

## MATERIALS AND METHODS

### Materials

Anti-mouse and anti-rabbit IgG-conjugated horseradish peroxidase, rabbit polyclonal antibody specific for β-actin was purchased from Santa Cruz Biotechnology (Santa Cruz, CA, USA). CCL5 and VEGF-C antibodies were purchased from Abcam (Cambridge, MA, USA). Recombinant human VEGF-C was purchased from R&D Systems (Minneapolis, MN, USA). Recombinant human CCL5 and the VEGF-C ELISA kit were purchased from PerpoTech (Rocky Hill, NJ, USA). The miR-507 mimic, miR-507 inhibitor, Lipofectamine 2000 and Trizol were purchased from Life Technologies (Carlsbad, CA, USA). The human miRNome microRNA Profilers QuantiMir™ kit was purchased from SBI (Mountain View, CA, USA). Dulbecco's modified Eagle's medium (DMEM), α-minimum essential medium (MEM), fetal bovine serum (FBS) and all other cell culture reagents were purchased from Gibco-BRL Life technologies (Grand Island, NY, USA). The dual-luciferase reporter assay kit was purchased from Promega (Madison, WI, USA). All other chemicals were purchased from Sigma-Aldrich (St Louis, MO, USA).

### Cell culture

The human chondrosarcoma cell line (JJ012) was kindly provided by the laboratory of Dr. Sean P. Scully (University of Miami School of Medicine, Miami, FL) and originated from Dr. Joel Block (Rush University Medical Center, Chicago, Illinois). The human chondrosarcoma cell line (SW1353) was purchased from the American Type Culture Collection (Manassas, VA, USA). Cells were cultured in DMEM/α-MEM supplemented with 10% FBS and 100 units/ml penicillin/streptomycin at 37°C with 5% CO_2_. The basal level of CCL5 in JJ012 cells is higher than in SW1351 cells (Supplementary Figure 5).

The human telomerase-immortalized human dermal lymphatic endothelial cells (hTERT-HDLECs), an immortalized human LEC line, was purchased from Lonza (Walkersville, MD, USA). These immortalized human LECs represent CD31 positive/podoplanin positive, and retain their ability to uptake acetylated LDL and induce tube formation. The human LECs were grown in EGM-2MV BulletKit Medium consisting of EBM-2 basal medium plus SingleQuots kit (Lonza). Cells were seeded onto 1% gelatin-coated plastic ware and cultured at 37°C with 5% CO_2_. We obtained the cryopreserved human LECs line from Lonza as passage 1, and maintained these cells according to manufacturer's instructions as well as used between passages 5 and 10 for experiments described herein.

### Generation of stable cell lines

Cells were seeded on plates and infected with pLKO_AS2.puro-vector, pLKO_AS2.puro-CCL5 or pLKO_1.puro-CCL5 shRNA by prepared lentivirus as described previously [28]. At 24 h after infection, stable clones were selected with puromycin. Thereafter, the selection medium was replaced every 2 days. After 2 weeks of selection, resistant clones were established.

### Preparation of conditioned medium

In the series of experiments, stable cell lines were incubated alone or transfected with miR-507 mimic or inhibitor for 24 h. After treatment, cells were washed and changed to serum-free medium. Conditioned medium (CM) was then collected 2 days after the change of medium and stored at −80°C until use.

### ELISA assay

The CM was used to determine the VEGF-C level in the medium of chondrosarcoma cells using VEGF-C ELISA kit (PerpoTech) according to the procedure described by the manufacturer.

### Transwell migration assay

Transwell inserts (8-μm pore size; Costar, NY, USA) in 24-well plates were used. The CM was collected from chondrosarcoma cells. LECs were seeded in the upper transwell chamber and 300 μL of CM was placed in the lower chamber. After 20 h, migrated cells were stained with crystal violet and counted under a microscope.

### Tube formation assay

Matrigel (BD Biosciences; Bedford, MA, USA) was dissolved at 4°C overnight, and 48-well plates were prepared with 150 μL Matrigel in each well after coating and incubating at 37°C for 30 min. LECs were resuspended at a density of 5 × 10^4^/100 μL in culture medium (50% EGM-2MV BulletKit Medium and 50% chondrosarcoma cell CM) and added to the wells. After 6 h of incubation at 37°C, LECs tube formation was assessed by microscopy, and each well was photographed. The number of tube branches and total tube length were calculated using the MacBiophotonics Image J software.

### Western blot analysis

The cellular lysates were prepared according our previous instruction [42]. The proteins were resolved using SDS-PAGE, then transferred to Immobilon polyvinyldifluoride (PVDF) membranes. Blots were blocked with 4% BSA for 1 h at room temperature, then probed with primary antibodies against VEGF-C (1:1000) and β-actin (1:2000) for 1 h at room temperature. After three washes, blots were subsequently incubated with the horseradish peroxidase-conjugated secondary antibody for 1 h at room temperature and visualized by enhanced chemiluminescence, using an Imagequant LAS 4000 (GE Healthcare, Pewaukee, WI, USA).

### Quantitative real-time PCR (qPCR) of mRNA and miRNA

Total RNA was extracted from cells as described previously [43]. The qPCR analysis was carried out using Taqman^®^ one-step PCR Master Mix (Applied Biosystems, CA, USA); 100 ng of total cDNA were added per 25 μl reaction with sequence-specific primers and Taqman^®^ probes. Sequences for all target gene primers and probes were purchased commercially (GAPDH was used as an internal control) (Applied Biosystems). qPCR assays were carried out in triplicate on a StepOnePlus sequence detection system. The cycling conditions consisted of 10-min polymerase activation at 95°C followed by 40 cycles at 95°C for 15 s and 60°C for 60 s. The threshold was set above the non-template control background and within the linear phase of the target gene amplification to calculate the cycle number at which the transcript was detected (denoted C_T_).

For the miRNA assay, cDNA was synthesized from total RNA (100 ng) using the TaqMan MicroRNA Reverse Transcription Kit (Applied Biosystems). Then, miRNome microRNA Profilers QuantiMir™ kit which contained 384 human miRNA primer was used to elucidate miRNA differential expression. The reactions were incubated first at 16°C for 30 min and then at 42°C for 30 min followed by inactivation at 85°C for 5 min, then incubated in a 96-well plate at 50°C for 2 min, 95°C for 10 min, followed by 30 cycles of 95°C for 15 s and 60°C for 60 s using the StepOnePlus sequence detection system. Relative gene expression was quantified using an endogenous control gene (U6). The threshold cycle (CT) was defined as the fractional cycle number at which fluorescence passed a fixed threshold, and relative expression was calculated using the comparative CT method.

### Plasmid constructs

The 3′-UTR-luciferase reporter constructs containing the 3′-UTR regions of VEGF-C with wild-type and mutant binding sites for miR-507 were amplified using the PCR method. The cDNAs were obtained from H293T cells. The PCR products were cloned into a *pmirGLO* reporter vector (Promega) between the *Nhe*I and *Xho*I restriction sites, instantly downstream of the luciferase reporter gene. The mutant 3′-UTRs were constructed by introducing seven mismatched mutations into putative seed regions of VEGF-C. All the constructs containing 3′-UTR inserts were sequenced and verified.

### Luciferase reporter assay

Cells were seeded on 6-well plates, then transiently transfected with VEGF-C 3′-UTR luciferase plasmids using Lipofectamine 2000 as described previously [44]. Cells collected were lysed with reporter lysis buffer 24 h after transfection, and the luciferase and renilla activities in the cellular extracts were determined by the dual-luciferase reporter assay kit. The relative luciferase activity was calculated by the ratio of luciferase/renilla activity, and normalized to that of control cells.

### Immunohistochemistry (IHC)

The human chondrosarcoma tissue array was purchased from CtbridTM (Rockville, MD, USA). The 9 cases for normal cartilage, 8 cases for type Ib chondrosarcoma (Grade I), 9 cases for type IIb chondrosarcoma (Grade II), and 12 cases for type IIIb chondrosarcoma (Grade III). The tissue slides were incubated and rehydrated in 3% hydrogen peroxide to block the endogenous peroxidase activity. After trypsinization, sections were blocked by incubation in 3% FBS in PBS. The primary antibody anti-human CCL5, VEGF-C or LYVE-1 was applied to the slides at a dilution from 1:50 to 1:150 and incubated at 4°C overnight [45]. After the slides were washed 3 times with PBS, the samples were treated with the secondary antibody at a dilution of 1:100. Bound antibodies were detected with an ABC kit (Vector Laboratories, Burlingame, CA). The samples were stained with chromogen diaminobenzidine, washed with PBS, counter-stained with Delafield's hematoxylin and dehydrated. Finally, the samples were treated with xylene and mounted. The intensity of staining was blindly evaluated by pathologist as 0, 1+, 2+, 3+, 4+, and 5+ for no staining, very weak staining, weak staining, moderate staining, strong staining, and very strong respectively. IHC score was determined as the sum of the intensity score.

### Tumor xenograft *in vivo* study

Four weeks old male nude mice were randomly divided into 3 groups (10 mice per group). For experimental cells growing exponentially, each implanted into mice by subcutaneous injection of 1 × 10^6^ human chondrosarcoma cells (JJ012/vector, JJ012/shCCL5 and JJ012/CCL5) were resuspended and mixed with Matrigel. After six weeks, mice were sacrificed and tumors were excised for IHC and q-PCR analysis, respectively. All procedures involving animal experiment were approved by the Institutional Animal Care and Use Committee at School of Medicine, China Medical University.

### Statistics

Data are presented as mean ± standard error of the mean (SEM). Statistical analyses of pairs of samples were performed using the Student's *t*-test. Statistical comparisons of more than two groups were performed using one-way analysis of variance (ANOVA) with Bonferroni's post-hoc test. In all cases, *p* < 0.05 was considered significant.

## SUPPLEMENTARY FIGURES


