# Supplementary material for: CCL5 promotes VEGF-C production and induces lymphangiogenesis by suppressing miR-507 in human chondrosarcoma cells
Source: Oncotarget. 2016 May 6;7(24):36896–908. doi: 10.18632/oncotarget.9213 (PMC5095047; doi:10.18632/oncotarget.9213)
Supplement: Supplementary file 1 [file oncotarget-07-36896-s001.pdf]

SUPPLEMENTARY FIGURES

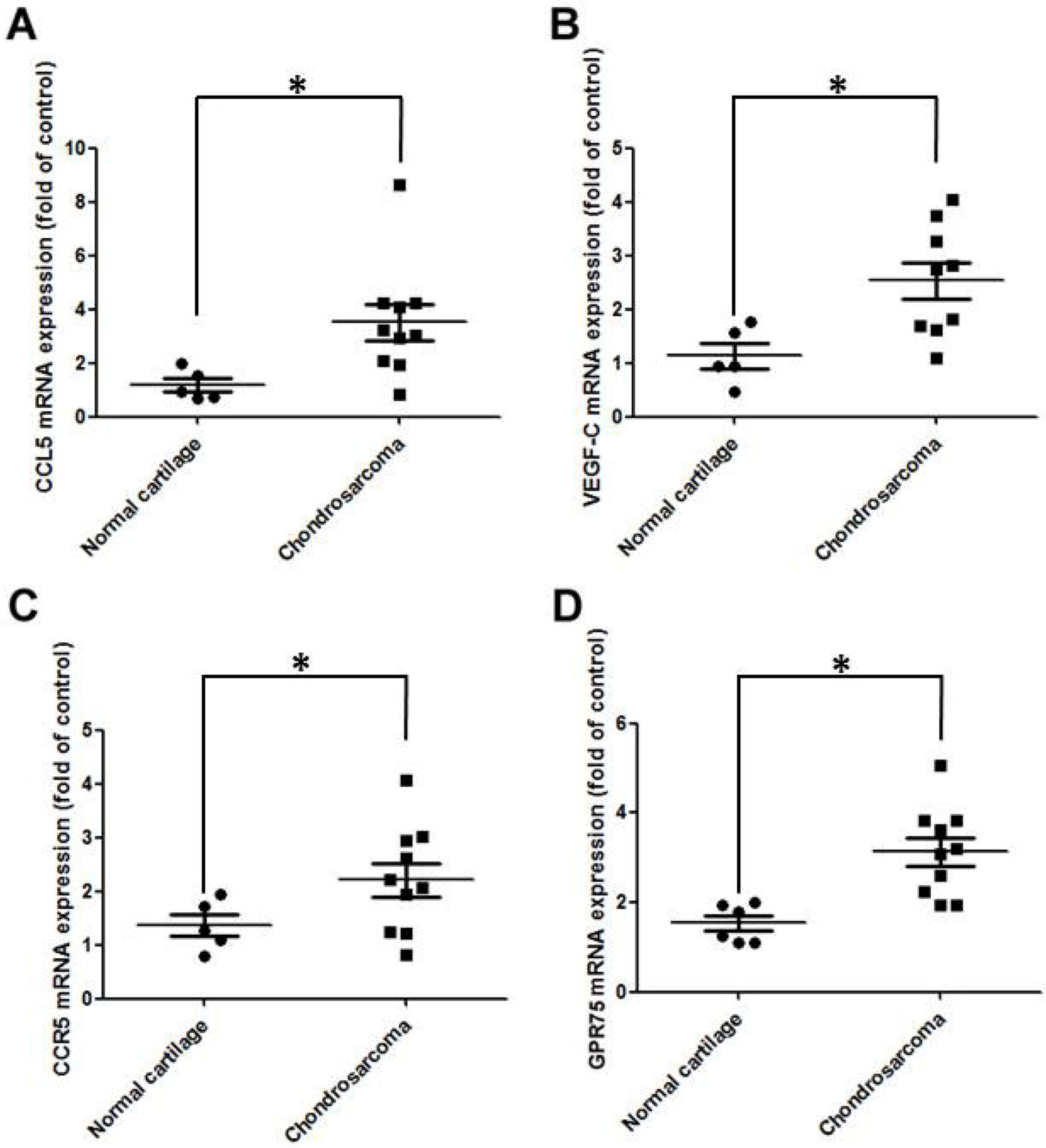

Supplementary Figure S1: Clinical significance of CCL5, VEGF-C, CCR5 and GPR75 expression in chondrosarcoma patients. The mRNA expression of CCL5, VEGF-C, CCR5 and GPR75 in normal cartilage (n=5) and chondrosarcoma patients (n=10) was examined by qPCR. Results are expressed as the mean ± SE. *, $p < 0.05$ compared with normal cartilage.

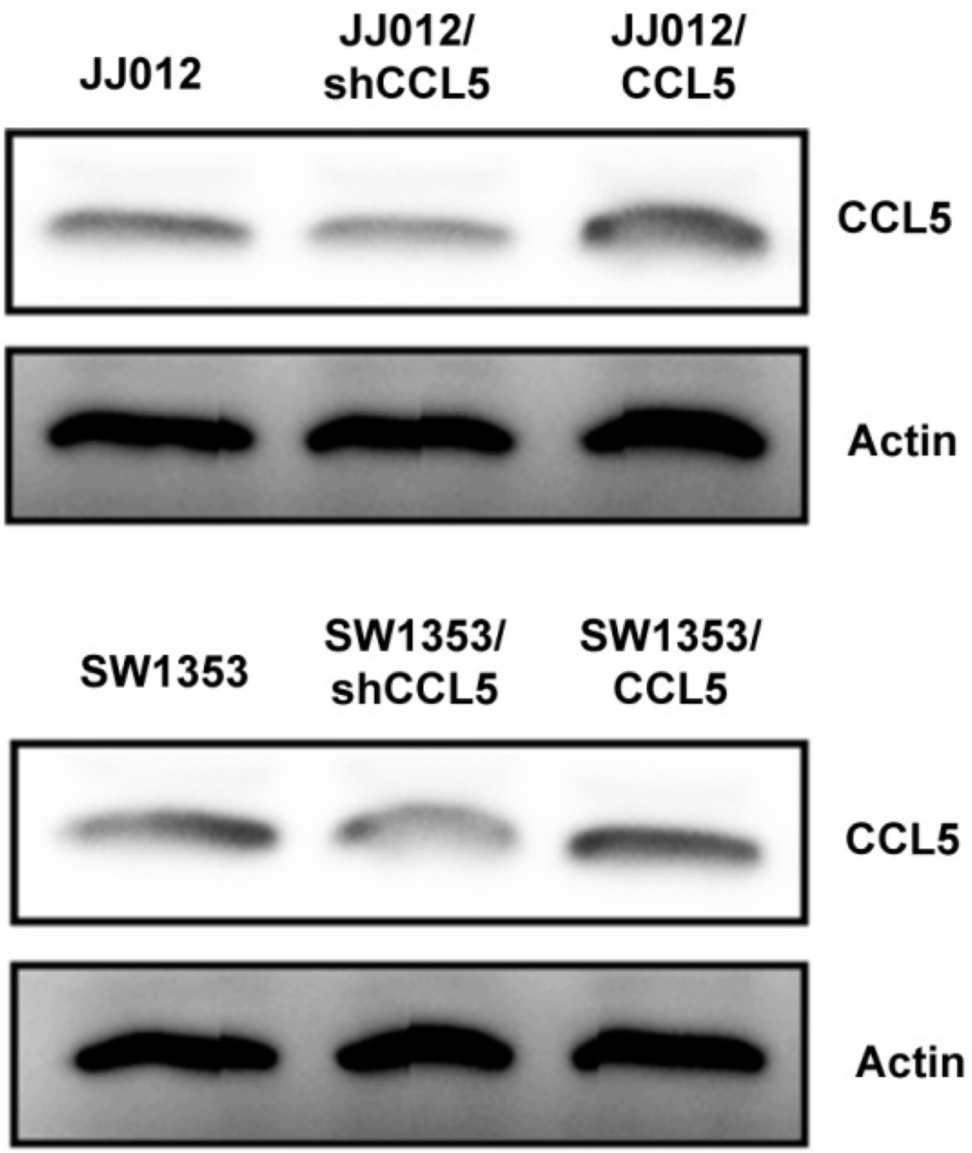

Supplementary Figure S2: CCL5 expression in CCL5-overexpressed clone, CCL5-knockdowned clone and vector control cells. The protein expression of CCL5 in the indicated chondrosarcoma cells was examined by Western blot.

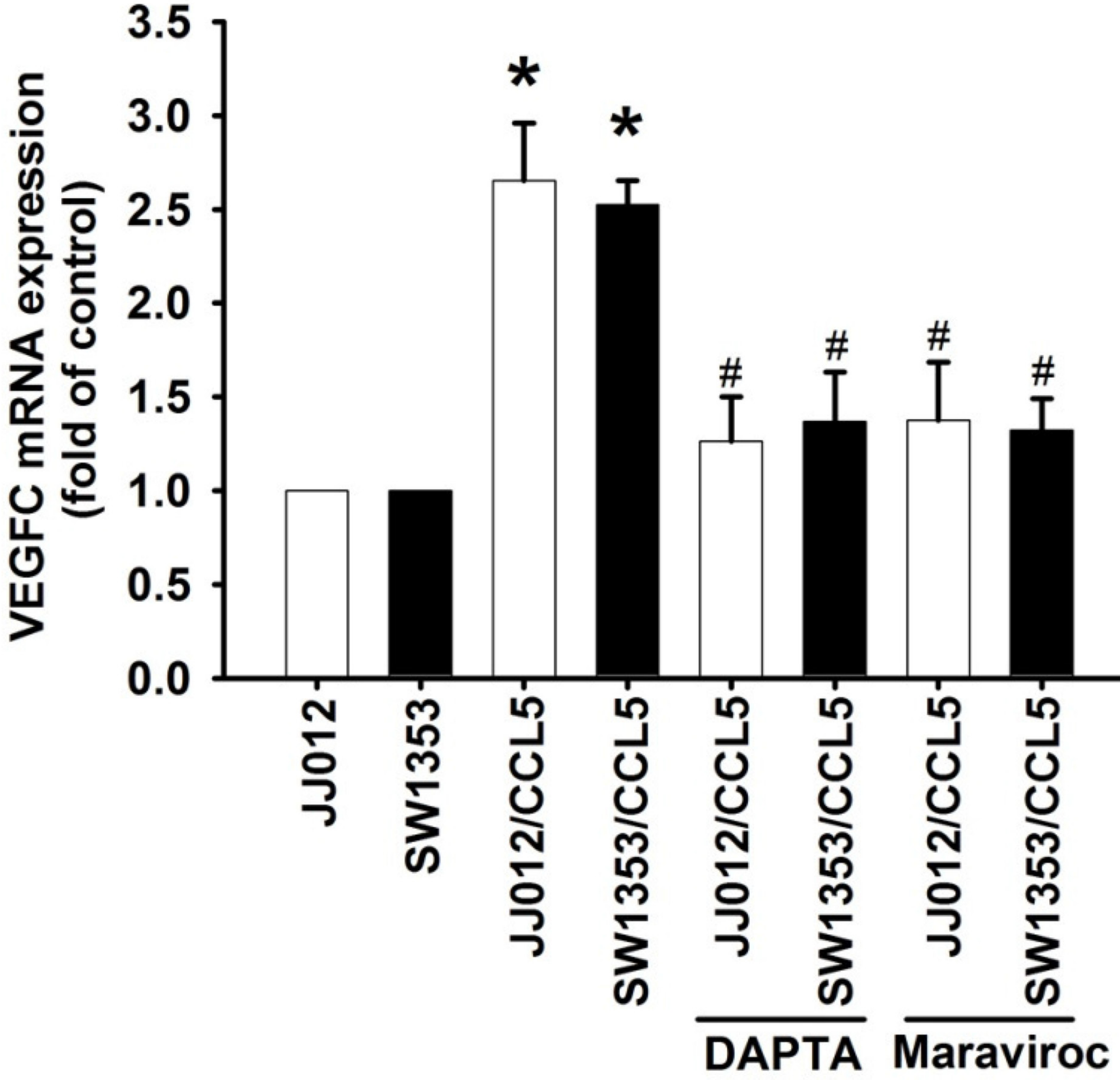

Supplementary Figure S3: Chemokine CCL5 promotes VEGF-C expression through CCR5 receptor. Cells were treated with DAPTA (10 nM) or maraviroc (100 nM) for 24 h. The VEGF-C mRNA expression was examined by qPCR. Results are expressed as the mean ± SE. *, $p < 0.05$ compared with control; #, $p < 0.05$ compared with CCL5-overexpressed group.

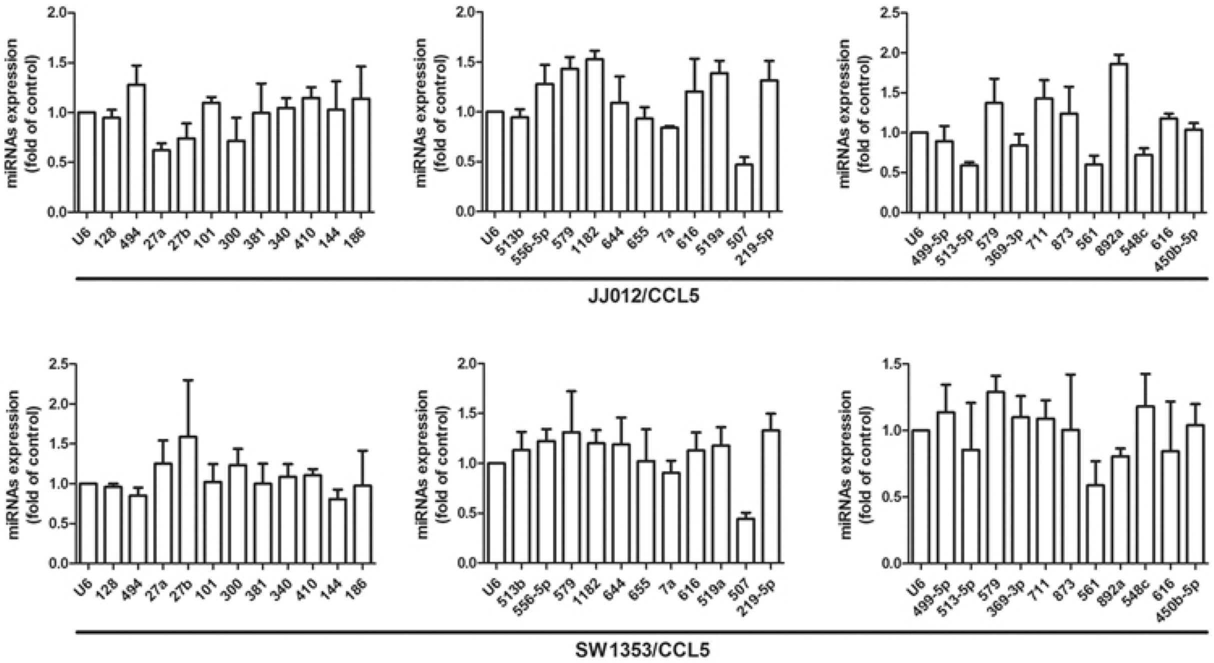

Supplementary Figure S4: Representative miRNA expression in CCL5-overexpressed chondrosarcoma cells. The JJ012/CCL5 and SW1353/CCL5 cells were cultured for miRNA analysis using qPCR. Results are expressed as the mean ± SE.

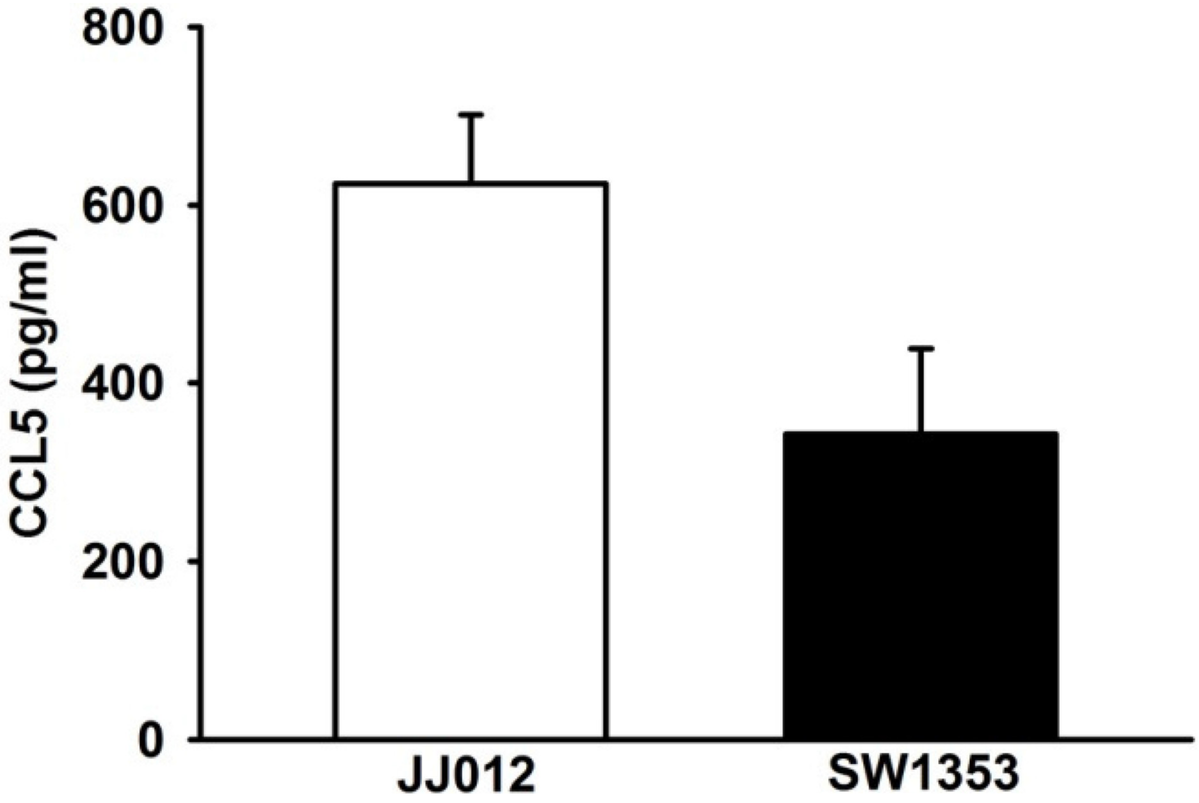

Supplementary Figure S5: The CCL5 expression in chondrosarcoma cell lines. JJ012 and SW1353 were cultured for 2 day. The CCL5 production in culture medium was examined by ELISA assay. Results are expressed as the mean ± SE.
